# Gender-based analysis of body dissatisfaction among youths in Singapore: findings from the National Youth Mental Health Study

**DOI:** 10.3389/fpsyt.2025.1505161

**Published:** 2025-04-25

**Authors:** Ellaisha Samari, Janhavi Ajit Vaingankar, Bernard Tan, Sherilyn Chang, Yeow Wee Brian Tan, S. Archana, Yi Chian Chua, Charmaine Tang, Yi Ping Lee, Chu Shan Elaine Chew, Courtney Davis, Swapna Verma, Mythily Subramaniam

**Affiliations:** ^1^ Research Division, Institute of Mental Health, Singapore, Singapore; ^2^ Department of Psychosis, Institute of Mental Health, Singapore, Singapore; ^3^ CHAT, Centre of Excellence in Youth Mental Health, Institute of Mental Health, Singapore, Singapore; ^4^ Adolescent Medicine Service, KK Women’s and Children’s Hospital, Singapore, Singapore; ^5^ SingHealth Duke-NUS Paediatric Academic Clinical Programme, Singapore, Singapore; ^6^ SingHealth Duke-NUS Global Health Institute, Singapore, Singapore; ^7^ Duke-NUS Medical School, Singapore, Singapore; ^8^ Saw Swee Hock School of Public Health, National University of Singapore, Singapore, Singapore; ^9^ Lee Kong Chian School of Medicine, Nanyang Technological University, Singapore, Singapore

**Keywords:** body dissatisfaction, gender-based analysis, Asia, BMI, social media use, childhood trauma, youth

## Abstract

**Introduction:**

Body dissatisfaction, often arising from the disparity between the perceived ideal and actual body, is prevalent among young individuals and is linked to various mental health issues. Previous research consistently indicates notable differences in body dissatisfaction between males and females. This study used data from a nationwide study to determine the prevalence of body dissatisfaction among young individuals in Singapore. It also aims to identify associated sociodemographic, health-related, and psychosocial factors using a gender-based approach.

**Methods:**

Data were obtained from 2600 youths aged 15-35 who participated in the National Youth Mental Health Study. Information on sociodemographic background, body dissatisfaction, childhood trauma, BMI, self-esteem, and social media use was collected. Weighted multiple logistic regressions were performed to identify the sociodemographic variables, as well as other health-related and psychosocial factors associated with body dissatisfaction, stratified by gender.

**Results:**

20.2% (25.7% females; 14.8% males) reported moderate to marked body shape concerns. Among females, younger ages, Indians (vs. Chinese), those with diploma education (vs. university), those with overweight, those with childhood trauma, and those with greater daily social media usage were more likely to report moderate to marked body shape concerns. Among males, those with primary education and below (vs. university), those with overweight, and those with greater daily social media usage were more likely to report moderate to marked body shape concerns.

**Discussion:**

These results emphasize the importance of creating specific public health programs that consider the different experiences and challenges related to body dissatisfaction based on gender. These programs could help promote body image positivity, increase self-compassion, and encourage critical engagement with social media content.

## 1 Introduction

Body image is a multifaceted construct encompassing perceptions, thoughts, feelings, attitudes, and behaviors related to one’s body (1, 2). A significant aspect of this construct is body dissatisfaction, characterized by a negative evaluation or subjective assessment of one’s body size and shape (3, 4). Body dissatisfaction is a prevalent issue affecting individuals worldwide (5). Various social agents, including the media, peers, and family, communicate powerful messages about the importance of appearance, which can pressurize individuals to conform to unrealistic body standards (6–8). When an individual perceives a discrepancy between the ideal body and their own body, it often results in body dissatisfaction (4).

Youth, characterized by rapid growth and transformative changes, represents a critical developmental period where an individual’s perception of their bodies becomes an important part of their self-concept and identity (9, 10). Unfortunately, research shows a troubling prevalence of body dissatisfaction among this demographic (11–13), which raises concerns due to its adverse effects on mental health and well-being. While numerous studies have explored body image issues, most have focused on college students, adolescents, clinical populations, and females in the United States and European countries, leaving limited research in Asian settings (13–15). Nonetheless, body dissatisfaction has been associated with a higher risk for various mental health conditions, such as eating disorders (16), depression, anxiety, and suicidal thoughts (12, 13, 17). Notably, the relationship between body dissatisfaction and mental health is complex, with some studies suggesting bidirectional effects. For example, research by Bornioli et al. (17) revealed that among females, body dissatisfaction predicted later depressive symptoms from adolescence to early adulthood. Conversely, among males, depressive symptoms predicted later body dissatisfaction in early adolescence and early adulthood, while the inverse relationship was more prominent during middle adolescence. Additionally, body dissatisfaction has also been associated with negative health behaviors such as alcohol and drug use, as well as engagement in risky behaviors such as self-harm (17, 18).

Research findings have shown that certain individuals are at a greater risk of experiencing body dissatisfaction. This includes individuals with obesity (19), those who have experienced childhood trauma (20), those with low self-esteem (21), and those who frequently compare their appearance to content on social media (22, 23). Notably, the existing body of research consistently indicates that females generally experience greater body dissatisfaction than males (24, 25).

There are significant differences in how females and males perceive and experience body image. In terms of body ideals, females tend to value a slender, toned, yet curvy physique, while men favor a muscular and lean body (6). Indeed, several studies have shown that female youths strive for a thinner body image (26) and are more likely to attempt to lose weight compared to their male counterparts (27). On the other hand, an increasing number of male youths are dissatisfied with their bodies, as many desire to be more muscular and taller (28, 29). Societal and cultural norms surrounding body image affect females and males differently. Research suggests that physical appearance holds greater significance for females than males (30). For females, physical appearance often plays a significant role in social comparison and self-evaluation (31), and thinness is seen as a valued attribute associated with success, status, and happiness in society (32). Sociocultural theories emphasize that the objectification of women in society, especially in Westernized contexts, contributes to increased body surveillance and self-objectification, which is often associated with poor body image (33). This phenomenon extends beyond Western cultures, where women in Asian countries like Japan and Taiwan face similar pressures surrounding thinness, mirroring patterns of body image concerns observed in Western societies (34).

Singapore presents a unique sociocultural landscape where body image is shaped by complex intersections of cultural influences. As a multiethnic, multilingual, and affluent society, the country experiences a dynamic mix of Western and East Asian beauty ideals. Nevertheless, unrealistic beauty ideals, such as a slim and fair-skinned appearance, dominate the media and social media platforms, which marginalize other body types and features and place disproportionate pressure on women (35, 36). Persistent weight-based stigmatization also remains a significant issue for individuals with obesity in Singapore (37).

Social media amplifies these sociocultural pressures, with algorithmic recommendations frequently exposing youths to body image-related content. A recent study found that 31% of Singaporean youths often encounter body image-related content, such as body checking and the body measurement challenge, highlighting the pervasive nature of these influences. These media-driven pressures not only reflect but actively reinforce narrow beauty standards, significantly contributing to body image concerns in Singapore (36).

This study addresses a significant geographical gap in body image research by examining the prevalence of body dissatisfaction and its associated factors among youths in Singapore using a nationally representative sample. Body dissatisfaction in this study was operationalized as ‘moderate to marked body shape concerns’ as assessed using the Body Shape Questionnaire-8C (BSQ-8C) (38), indicating a persistent and significant preoccupation with body shape. This exploratory study aims (1) to assess the overall prevalence of body dissatisfaction, including gender-specific rates among male and female youths, and (2) to explore the factors associated with body dissatisfaction within each gender. Accordingly, the research seeks to answer: *What is the prevalence of body dissatisfaction among youths in Singapore, and how does it vary by gender?* and *Which factors are associated with body dissatisfaction in males and females, respectively?*

## 2 Materials and methods

### 2.1 Study design

The data used in this study were obtained from the National Youth Mental Health Study, a nationwide cross-sectional epidemiological study conducted among Singapore citizens and permanent residents aged between 15 and 35. The purpose of the study was to determine the extent of critical mental health issues and their associations among young people in Singapore. For the current study, we specifically analyzed data related to body dissatisfaction and mental health from this larger cohort, focusing on sociodemographic background, body dissatisfaction, childhood trauma, BMI, self-esteem, and social media use.

### 2.2 Sample population

The study employed a convenience sampling approach with multi-site recruitment (39). Participants were selected disproportionately across various geographical districts in Singapore based on gender, age groups (15–19, 20–24, 25–29 and 30–35), and ethnicity. This approach was adopted to ensure a sufficient sample size within each subgroup, thereby increasing the reliability of subgroup analyses.

Participants were recruited from two primary sites: residential areas and public spaces. In residential areas, household-level sampling was conducted by randomly generating postal codes and approaching individuals at their residences. This method ensured systematic recruitment across various neighborhoods, contributing to the geographic diversity of the sample. To complement this approach and include hard-to-reach groups, such as youths in National Service (NS) and youths in the younger age groups, a street intercept method was employed. Interviewers targeted public spaces and high-traffic locations where these groups were likely to be present, such as transportation hubs, shopping malls, outside schools, and recreational areas. For youths in NS, we focused on public spaces where NS personnel were likely to be found during off-duty hours, such as weekends.

Individuals were eligible to participate if they were Singapore citizens or permanent residents, aged 15-35 years, literate in English, Mandarin, Malay, or Tamil, and able to provide written informed consent. For participants under 21 years of age, consent was also obtained from a legally acceptable representative. Youths who were unable to complete the survey on their own and those who were unable to provide written consent were excluded from the study.

The required sample size was determined using power calculations to ensure precise estimates of mental health prevalence with a 5% margin of error, 80% statistical power, and a 5% Type 1 error rate. Prevalence estimates were based on the Singapore Mental Health Study 2016, where mental disorders among youths aged 18-34 ranged from 2.2% (generalized anxiety disorder) to 9.2% (major depressive disorder) (40). An estimated design effect (DEFF) of 1.83, accounting for oversampling by age and ethnicity was incorporated into the calculations. A target sample of 2500 was deemed sufficient for precision, with an additional 100 participants recruited to account for potential missing data, resulting in a final sample of 2600 youths.

### 2.3 Data collection

Data were collected between October 2022 and June 2023 through a self-administered survey, conducted by trained interviewers. The survey was available in four languages (English, Mandarin, Malay, and Tamil) to ensure accessibility for all participants.

The survey was administered in various private and public settings, such as the participants’ homes or the void deck in public housing areas, with careful consideration given to their convenience. To ensure privacy and comfort, participants were provided with a tablet to complete the survey independently in quiet, low-traffic areas where they could focus without distractions. The timing of the survey was flexible to accommodate participants’ schedules, and interviewers were available to assist with any queries, ensuring minimal bias while allowing participants to complete the survey autonomously.

Interviewers emphasized the confidentiality of participants’ responses and encouraged participants to complete them in a private space, away from potential distractions or influence from family members, to reduce potential influences such as social desirability bias. Upon completion, participants were compensated for their time and effort.

#### 2.3.1 Questionnaires

##### 2.3.1.1 Sociodemographic information

Sociodemographic information, including age, gender, ethnicity, marital status, education level, employment status, and monthly household income, was obtained. Gender was categorized as ‘male’ or ‘female’ based on participants’ self-reports. Employment status was categorized as ‘employed’ (including both full-time and part-time workers), ‘unemployed’ and ‘economically inactive’ (i.e., homemakers and students who are not working).

##### 2.3.1.2 Body dissatisfaction

The short version of the Body Shape Questionnaire (BSQ-8C) (38): The BSQ-8C is the short version of the Body Shape Questionnaire (BSQ), which consists of eight items obtained from the full version of 34 items. These eight items measure concerns with body shape in the past four weeks (e.g., ‘Have you been afraid that you might become fat (or fatter)?’, ‘Has thinking about your shape interfered with your ability to concentrate?’, ‘Has seeing your reflection made you feel bad about your shape?’). The scale uses a 6-point scale ranging from 1 (never) to 6 (always). Total scores are calculated, with higher scores indicating greater body shape dissatisfaction. Scores are categorized as follows: No concern: score < 19 (indicates minimal preoccupation with body shape); Mild concerns: score 19-25 (suggests some occasional negative thoughts about body shape); Moderate concerns: score 26-33 (reflects persistent and more frequent negative thoughts about body shape); and Marked concerns: score >33 (indicates significant and pervasive preoccupation with body shape that likely substantially interferes with psychological well-being). For analytical purposes, this study grouped moderate (scores 26-33) and marked concerns (scores >33) into a single category of moderate to marked body shape concerns. This approach allowed for a comprehensive analysis of factors associated with elevated body dissatisfaction. In this study, body dissatisfaction was operationalized as having moderate to marked body shape concerns.

The BSQ-8C was chosen for this study due to its brevity and strong psychometric properties, making it well-suited for large-scale, population-based surveys where minimizing participant burden is essential. Unlike longer measures such as the full BSQ (41), the BSQ-8C offers an efficient assessment without compromising reliability or validity. Furthermore, the scale has demonstrated excellent internal consistency and validity across diverse populations, including youth (38, 42), and shows high sensitivity to changes over time – an important feature for both clinical and research settings (43). In our sample, the BSQ-8C exhibited excellent internal consistency, with a Cronbach’s alpha of 0.950 for the overall sample, 0.953 for females, and 0.941 for males. These values are consistent with prior studies, further confirming the scale’s reliability in youth populations.

##### 2.3.1.3 Health-related and psychosocial factors associated with body dissatisfaction

The Adverse Childhood Events – International Questionnaire (ACE-IQ) (44) : The ACE-IQ was used to ascertain whether participants had experienced any abuse or neglect during their first 18 years of life. Specifically, it includes questions about physical, sexual, and emotional abuse and neglect by parents or caregivers; peer violence; living with household members who were alcohol and/or drug users or incarcerated; violence against household members; having one or no parents, parental separation, or divorce; witnessing community violence; and exposure to collective violence. For this study, data on community violence and exposure to collective violence were not included as they are relatively uncommon in Singapore. Specifically, 8.1% (n=212) of youths reported ever seeing or hearing someone being stabbed or shot and 10.5% (n=274) reported being threatened with a knife or gun. However, 46.9% (n=1220) reported witnessing someone being beaten up. Regarding exposure to collective violence, 3.7% (n=95) of youths reported ever being forced to relocate, 3.1% (n=81) witnessed the deliberate destruction of their homes, and 2.4% (n=62) were beaten up by soldiers, police, militia, or gangs. Additionally, 2.3% (n=59) reported having a family member or friend killed by these groups. Given the low prevalence of most of these experiences and their limited correlation with body dissatisfaction, these data were not included in the analysis. Each type of ACE exposure was assessed with a binary measure of ‘yes’ or ‘no’, and the frequency of each exposure was measured as ‘many times’, ‘a few times’, ‘once’, or ‘never’. Frequency scoring was used in this study, whereby the frequency of an ACE experience is considered for the item to be scored (45). Responses were then subsequently categorized as ‘no ACE’ (i.e., no experience of ACE) and ‘1 or more ACE’ (i.e., experiencing one or more types of ACE) for analysis. This cut point of at least one ACE allows for a sensitive exploration of potential associations with body dissatisfaction, recognizing that even minimal exposure to childhood adversity may be associated with body image experiences. Furthermore, based on the findings of the recent Singapore Mental Health Study, the presence of even once ACE was significantly associated with increased odds of adverse mental health outcomes (46). The Cronbach’s alpha was 0.760 for the overall sample, 0.751 for the female sample, and 0.783 for the male sample.

Rosenberg Self-Esteem Scale (RSE) (47): The RSE scale is a ten-item self-report questionnaire. It measures both positive and negative feelings related to self-worth and self-esteem. Each item is answered on a 4-point Likert scale, from 1 (strongly disagree) to 4 (strongly agree). Negatively worded items are reversed in valence. Total scores are calculated, with higher scores indicating higher self-esteem. The Cronbach’s alpha was 0.855 for the overall sample, 0.854 for the female sample, and 0.854 for the male sample.

Body Mass Index (BMI): Participants’ BMI (kg/m^2^) was calculated using their self-reported weight (in kilograms) and height (in meters) and classified according to the World Health Organization standards: Underweight (BMI < 18.5), Normal weight (BMI = 18.5 – 24.9), Pre-obesity (BMI = 25.0 – 29.9), and Obese (BMI ≥ 30.0) (48). For this paper, the term ‘overweight’ is used instead of ‘pre-obesity’ for ease of comparison with the literature.

Social media use: Participants were asked to indicate their daily usage of social media platforms, selecting from the following time intervals: less than 30 minutes, more than 30 minutes but less than an hour, one to two hours, two to three hours, three to four hours, four to five hours, five to six hours, six to seven hours, or more than seven hours. For analysis, the responses for ‘less than 30 minutes’ and ‘more than 30 minutes but less than an hour’ were combined and represented as ‘up to one hour’. These responses were treated as ordinal variables, with each interval indicating an increase (additional hour) in usage duration.

### 2.4 Statistical analyses

Post-stratification weighting was applied to the sample using data from the 2022 Singapore population census based on three key demographic variables: age group, gender, and ethnicity. This was done to ensure that all analyses represented the Singapore youth population and that the findings were generalizable.

Descriptive statistics were conducted to determine the prevalence of body dissatisfaction in this sample. Following this, weighted binary multiple logistic regressions were performed to identify the sociodemographic variables (i.e., age, ethnicity, marital status, household income and employment status) and other factors (i.e., adverse childhood events, self-esteem, BMI, and social media usage) associated with body dissatisfaction (defined as moderate to marked body shape concerns). Analyses were conducted for both the overall sample and stratified by gender to explore potential gender-specific associations. All independent variables were entered simultaneously into the models based on theoretical relevance and previous research, allowing for adjustment of potential confounding effects.

Statistical significance was set at p<0.05. All statistical analyses for this study were carried out using the IBM SPSS statistics version 23 for descriptive statistics and SAS for applying post-stratification weights and conducting regression analysis.

## 3 Results

### 3.1 Sample characteristics

Two thousand six hundred individuals participated in the survey. Approximately half of the sample were female (50.2%). The majority of the participants were Chinese (71.5%), single (74.1%) and employed (62.3%). Among those employed, most (92.3%) were working full-time. **Table 1** displays the sociodemographic distribution of the sample.

**Table 1:** Sociodemographic characteristics of the overall sample.

	N	Weighted %
Age		
15-19	632	18.8
20-24	672	21.2
25-29	634	25.4
30-35	662	34.6
Gender		
Female	1219	50.2
Male	1381	49.8
Ethnicity		
Chinese	1313	71.5
Malay	658	16.4
Indian	506	9.1
Others**^†^**	123	3.1
Education level		
Primary and below	182	5.1
Secondary	520	16.4
Pre-U/Junior College	260	8.7
Vocational/Technical diploma	314	9.4
Polytechnic/Other diploma	681	25.5
University and above	643	34.8
Marital status		
Single	2006	74.1
Married	557	24.6
Separated/ Divorced/ Widowed	37	1.4
Monthly household income		
Less than SGD5,000	974	31.4
SGD5,000 - 9,999	859	33.8
SGD10,000-19,999	550	25.0
More than SGD20,000	217	9.8
Employment status		
Unemployed	166	6.4
Employed^^^	1461	62.3
Economically inactive*	973	31.3

**^†^** Others: Arab, Burmese, Caucasian, Eurasian, Filipino, Vietnamese, and more.

^Employed: Includes individuals who are working full-time or part-time.

*Economically Inactive: Homemakers and students who are not working.

### 3.2 Weighted prevalence of body dissatisfaction

22.7% (n=517) of young people in Singapore reported mild concerns about their body shape in the past four weeks, while 20.2% (n=575) reported moderate to marked concerns. When comparing across genders, 25.7% of females and 14.8% of males expressed moderate to marked body shape concerns. Notably, individuals aged 20-24 (25.2%) exhibited the highest prevalence of moderate to marked body shape concerns. **Table 2** provides further insight into the prevalence of moderate to marked body shape concerns across various sociodemographic groups.

**Table 2:** Prevalence of moderate to marked body shape concerns.

	Overall sample		Females		Males	
	N	Weighted %	N	Weighted %	N	Weighted %
Overall prevalence	575	20.2	353	25.7	222	14.8
Age						
15-19	156	23.5	114	35.4	42	12.0
20-24	163	25.2	73	32.0	90	18.8
25-29	150	22.6	97	27.4	53	17.7
30-35	106	13.7	69	15.8	37	11.5
Ethnicity						
Chinese	232	17.6	128	22.4	104	12.9
Malay	183	26.9	120	33.7	63	20.4
Indian	126	25.7	85	34.3	41	17.1
Others**^†^**	34	28.8	20	33.4	14	22.4
Education						
Primary and below	48	25.2	33	33.7	15	16.8
Secondary	135	25.6	94	35.3	41	15.9
Pre-U/Junior College	50	17.7	22	22.4	28	14.0
Vocational/Technical diploma	74	24.5	35	32.0	39	19.7
Polytechnic/Other diploma	155	20.4	88	24.2	67	16.7
University and above	113	16.3	81	20.9	32	10.6
Marital status						
Single	461	21.5	271	28.5	190	15.1
Married	106	16.3	77	18.7	29	13.0
Separated/ Divorced/ Widowed	8	24.6	5	25.7	3	22.9
Monthly household income						
Less than SGD5,000	226	21.9	137	27.9	89	16.2
SGD5,000 - 9,999	190	19.9	115	23.8	75	15.9
SGD10,000-19,999	110	18.1	70	22.8	40	12.6
More than SGD20,000	49	21.7	31	33.5	18	11.7
Employment status						
Unemployed	35	18.2	22	19.4	13	16.7
Employed^^^	303	18.5	175	22.9	128	14.5
Economically inactive*	237	24.1	156	31.9	81	15.1

**^†^** Others: Arab, Burmese, Caucasian, Eurasian, Filipino, Vietnamese, and more.

^^^Employed: Includes individuals who are working full-time or part-time.

*Economically Inactive: Homemakers and students who are not working.

### 3.3 Association between body dissatisfaction and sociodemographic variables

After adjusting for sociodemographic variables and other factors (adverse childhood events, self-esteem, BMI, and social media usage), females were more likely (OR=2.77; 95% CI=2.12-3.60) to report moderate to marked body shape concerns than males.

Among females, age, ethnicity, education level, and employment status were significantly associated with moderate to marked body shape concerns. Specifically, those in the age groups of 15-19, 20-24, and 25-29 were 4.26 (95% CI=1.82-9.97), 3.40 (95% CI=1.78-6.51), and 2.04 (95% CI=1.27-3.29) times more likely than those in the 30-35 age group to report moderate to marked body shape concerns, respectively. Those of Indian ethnicity (vs. Chinese) (OR=2.00; 95% CI=1.27-3.17) were more likely to report moderate to marked body shape concerns, while those with diploma qualification (vs. university) (OR=0.54; 95% CI=0.31-0.92) and those who were unemployed (vs. employed) (OR=0.27; 95% CI=0.11-0.68) were less likely to report moderate to marked body shape concerns (**Table 3**).

**Table 3:** Sociodemographic, health-related and psychosocial factors associated with moderate to marked body image concerns, stratified by gender.

	Females			Males		
	OR	95% CI	p-value	OR	95% CI	p-value
Age groups						
15-19	4.26	1.82 - 9.97	**<0.001**	0.78	0.35 - 1.73	0.538
20-24	3.40	1.78 - 6.51	**<0.001**	1.82	0.95 - 3.47	0.071
25-29	2.04	1.27 - 3.29	**0.003**	1.43	0.80 - 2.56	0.227
30-35	Reference			Reference		
Ethnicity						
Indian	2.00	1.27 - 3.17	**0.003**	1.37	0.82 - 2.31	0.234
Malay	1.12	0.71 - 1.76	0.637	1.09	0.67 - 1.76	0.739
Others	1.86	0.81 - 4.27	0.141	1.44	0.67 - 3.12	0.352
Chinese	Reference			Reference		
Education						
Primary and below	0.64	0.23 - 1.76	0.386	2.91	1.02 - 8.33	**0.047**
Secondary	0.80	0.36 - 1.78	0.581	1.36	0.63 - 2.96	0.435
Vocational/ ITE	0.39	0.17 - 0.93	**0.033**	1.26	0.62 - 2.53	0.524
Pre-u/ JC	0.90	0.43 - 1.87	0.767	1.13	0.56 - 2.25	0.739
Diploma	0.54	0.31 - 0.92	**0.022**	0.91	0.48 - 1.72	0.764
University	Reference			Reference		
Marital status*						
Single	0.97	0.60 - 1.57	0.915	1.05	0.58 - 1.90	0.873
Married	Reference			Reference		
Monthly Household income						
SGD5,000 - 9,999	1.16	0.74 - 1.81	0.518	1.38	0.86 - 2.19	0.179
SGD10,000 - 19,999	1.15	0.70 - 1.86	0.587	1.16	0.67 - 2.01	0.603
more than SGD20,000	1.84	0.91 - 3.75	0.092	1.20	0.59 - 2.44	0.620
Less than SGD5,000	Reference			Reference		
Employment status						
Unemployed	0.27	0.11 - 0.68	**0.005**	0.70	0.29 - 1.72	0.437
Economically inactive	0.95	0.53 - 1.70	0.858	1.19	0.77 - 1.85	0.437
Employed	Reference			Reference		
BMI (kg/m^2^)						
Underweight (<18.5)	0.26	0.15 - 0.46	**<0.001**	0.20	0.07 - 0.56	**0.002**
Overweight (25.0-29.9)	2.81	1.75 - 4.49	**<0.001**	4.09	2.60 - 6.45	**<0.001**
Obese (≥30)	2.89	1.60 - 5.23	**<0.001**	8.44	5.05 - 14.08	**<0.001**
Normal weight (18.5-24.9)	Reference			Reference		
Adverse Childhood Events						
Yes	2.22	1.51 - 3.25	**<0.001**	1.38	0.92- 2.08	0.123
No	Reference			Reference		
Self-esteem	0.79	0.75 - 0.84	**<0.001**	0.85	0.81 - 0.88	**<0.001**
Social media use^†^	1.14	1.05 - 1.25	**0.003**	1.17	1.06 - 1.29	**0.002**

*Youths who were divorced, separated, or widowed (N=37) were not included in analysis due to small sample size. ^†^Per hour increase. Bold values indicate statistical significance (p<0.05).

On the contrary, only education was significantly associated with moderate to marked body shape concerns among males. Specifically, those with primary and below qualification (vs. university) (OR=2.91; 95% CI=1.02-8.33) were more likely to report moderate to marked body shape concerns (**Table 3**).

### 3.4 Association between body dissatisfaction and other health-related and psychosocial factors

Among females, BMI, adverse childhood events, self-esteem, and daily social media usage were significantly associated with moderate to marked body shape concerns. Compared to those with normal weight, individuals with overweight or obesity were 2.81 (95% CI=1.75-4.49) and 2.89 (95% CI=1.60-5.23) times more likely to exhibit moderate to marked body shape concerns, respectively. Conversely, those with underweight (vs. normal weight) were (OR= 0.26; 95% CI=0.15-0.46) less likely to report such concerns. Higher self-esteem (OR=0.79; 95% CI=0.75-0.84) was associated with a lower likelihood of reporting moderate to marked body shape concerns. Experiencing at least one adverse childhood event (vs. none) (OR=2.22; 95% CI=1.51-3.25) and higher daily social media usage (OR=1.14; 95% CI=1.05-1.25) were associated with a greater likelihood of experiencing moderate to marked body shape concerns (**Table 3**).

Among males, BMI, self-esteem, and daily social media usage were significantly associated with moderate to marked body shape concerns. Compared to those with normal weight, individuals with overweight or obesity were 4.09 (95% CI=2.60-6.45) and 8.44 (95% CI=5.05-14.08) times more likely to exhibit moderate to marked body image concerns. Conversely, those with underweight (vs. normal weight) were less likely to report such concerns (OR=0.20; 95% CI=0.07-0.56). Higher self-esteem (OR=0.85; 95% CI=0.81-0.88) was associated with a lower likelihood of reporting moderate to marked body shape concerns. Higher daily social media usage (OR=1.17; 95% CI=1.06-1.29) was associated with a greater likelihood of reporting moderate to marked body shape concerns (**Table 3**).

## 4 Discussion

This study contributes to the growing body of literature on body dissatisfaction, particularly in the context of youths in Asia, and provides valuable insights into its prevalence within the Singaporean context. In this study, 20.2% experienced moderate to marked body shape concerns. This percentage is lower compared to studies conducted among youths in Australia (29.0%) and university students in Malaysia (40%) and Sweden (22%) (11, 12, 49). Among females, 25.7% reported moderate to marked body shape concerns, which is lower than the rates observed among female youths in Australia (46.2%) and India (31%) (11, 50). For males, 14.8% reported moderate to marked body shape concerns, which is lower than the prevalence among male youths in India (22%) but slightly higher than male youths in Australia (13.4%) (11, 50). While these findings suggest that body dissatisfaction among youths in Singapore may be less prevalent than in some Western and regional counterparts, the persistence of concerns across gender groups highlight the need for body image and mental health interventions. Given the established associations between body dissatisfaction and mental health outcomes, such as low self-esteem, disordered eating, and depressive symptoms, addressing body image concerns among youths in Singapore is critical for public health efforts. However, cross-study comparisons should be interpreted with caution due to variations in measurement scales and reporting methods (51).

The observed gender differences in body dissatisfaction are consistent with previous research, showing that body dissatisfaction is more pronounced among females compared to males. This disparity may stem from the strong societal emphasis on female beauty, particularly in traditional media and on social media (35, 52). In the local context, teenage girls in Singapore have been shown to deeply internalize narrow beauty standards displayed on social media platforms, that focus intensely on features such as bright eyes, flawless skin, and thinness. These beauty standards are further reinforced through self-presentation practices, where edited selfies and the pursuit of likes and followers serve as tools for negotiating social acceptance (52). Moreover, social media pressures in Singapore are further compounded by unique cultural influences, such as the popularity of K-pop and East Asian beauty ideals, which may further exacerbate concerns about appearance. The prominence of social media influencers who endorse unnecessary beauty treatments and cosmetic enhancements in Singapore could also contribute to unattainable beauty standards, intensifying body dissatisfaction among young females. Given these multiple layers of influence – from social media to cultural beauty standards – our findings emphasize the need for culturally tailored public health interventions that address the pervasive impact of social media and promote body diversity, particularly among young women navigating these societal pressures.

Beyond these broader cultural influences, our analysis revealed important differences in demographic patterns in body dissatisfaction among females and males. Specifically, age, ethnicity, and employment were significantly associated with moderate to marked body shape concerns in females but not males, suggesting gender-specific influences. Notably, female youths aged 15-19, 20-24, and 25-29 were more likely to experience moderate to marked body image concerns than those aged 30-35. This pattern aligns with previous research indicating that early youth is generally marked by greater pressure to conform to societal ideals and more frequent engagement in peer comparisons (53–56). Our findings extend this understanding within Singapore’s unique cultural context. Additionally, physical changes that occur in girls during puberty, such as increased weight, may further amplify these pressures by creating a greater gap between actual and ideal body image (10). These findings highlight how age-specific vulnerabilities interact with broader sociocultural influences, suggesting that interventions may need to be particularly targeted toward younger female demographics while addressing the unique pressures they face in Singapore’s social context.

This study also highlights cultural nuances that diverge from findings in other regions. One notable example is the association between unemployment and body dissatisfaction in females. While a Spanish study found that unemployed women were more concerned with their appearance than employed women (57), our findings suggest the opposite. Unemployed female youths reported a significantly lower likelihood of experiencing moderate to marked body shape concerns than employed female youths. This dissimilarity may reflect cultural differences in societal expectations regarding appearance, particularly in relation to employment. The pressure to maintain a “slender” body type in Singapore might be especially salient in professional settings, where appearance could influence hiring decisions, promotions, or workplace treatment. Supporting this, a study in Singapore among individuals with obesity found that a significantly higher number of respondents with class III obesity reported experiencing weight stigma, such as missing out on jobs, being overlooked for promotions, or being retrenched due to their weight. However, the study did not investigate the differential impact across genders (37). It is also important to consider that the association observed between unemployment and body dissatisfaction may be influenced by the smaller sample size of unemployed female youths in our study, which could affect the precision and reliability of these findings. Additionally, the age group in our study – youths – may partially explain the differences in results, as body image concerns can vary significantly between youths and adults (57).

Cultural variations in appearance-related expectations within the workplace have also been observed in other Asian contexts. For example, recent research in China showed that women experience distinct body shape pressures in the workplace, with workplaces being more accepting of male body types than female ones and women more likely to face bias or discrimination based on their weight appearance (57). These findings suggest that body dissatisfaction in relation to employment is shaped by broader cultural and societal norms, reinforcing the importance of considering local workplace dynamics when addressing body image concerns.

Our study also builds on earlier research exploring the relationship between education level and body dissatisfaction in males. While studies have yielded mixed results in the past (58–60), we found that males with lower education levels were more likely to experience moderate to marked body shape concerns. This prompts further research into how educational attainment may influence body image perceptions among male youths, which could be important in understanding the broader factors that shape body dissatisfaction, especially given the limited research on this demographic.

Building on these demographic findings, the association between other health-related and psychosocial factors (i.e., BMI, self-esteem, and social media usage) and body dissatisfaction appears consistent across both genders. Specifically, individuals with overweight or obesity, regardless of gender, were more likely to experience moderate to marked body image concerns compared to those within the normal weight range – a pattern that has been consistently observed across various populations in previous research (19). Societal norms and media portrayals often idealize slenderness, influencing individuals’ perceptions and evaluations of their physical appearance (61). The pressure from society to achieve the "ideal" slender body type can lead to greater body dissatisfaction among youths with overweight or obesity. Furthermore, this pressure associates slenderness with the strength of will and self-control, leading to significant stigma and body shaming for those who do not fit this ideal, potentially exacerbating dissatisfaction (62, 63). Unfortunately, weight stigma is prevalent among youths with high body weight in many countries, including Singapore (37, 64).

Conversely, both female and male youths classified as underweight were less likely to experience moderate to marked body image concerns compared to those in the normal weight range. A study conducted in Poland found similar results for adolescent girls, while underweight adolescent boys were found to have similar levels of body satisfaction to their normal-weight counterparts (65). In contrast, Dion et al. (66) found that among adolescents in Canada, boys tended to be dissatisfied with their shape when their BMI was below or above average, whereas girls tended to be dissatisfied when their BMI was average or above average. Our finding that underweight girls had lower dissatisfaction scores compared with normal-weight girls likely relates to the prevailing societal ideal of a thin body among females (6). Furthermore, prior studies have shown that many adolescent girls and young female adults desire to be thinner, even among those within the normal weight range (66). Interestingly, body dissatisfaction was lower among underweight males than normal-weight males, which contradicts typical body norms for males that favor a lean and muscular physique (6). However, Monocello’s research indicates that dominant masculinity, characterized by strong, bulk muscles, is not universally embraced (67). The study revealed that young people in South Korea often admire men with “smaller muscles,” referring to slim, defined muscles associated with swimmers, models, and K-Pop idols. This body type is seen as representing cultural sophistication and social appropriateness, as portrayed by K-pop idols and actors. The results of our study indicate the importance of better understanding body satisfaction among underweight and normal-weight individuals in this population and identifying potential cultural nuances in body norms and perceptions, particularly among males.

In addition, individuals who spend more time on social media daily, regardless of gender, were more likely to experience moderate to marked body image concerns. This aligns with previous research that has shown a strong correlation between social media usage and body dissatisfaction among young people (23, 68–71), particularly when users engage in appearance comparisons, which can negatively affect their self-perception of beauty (72–74). A recent survey in Singapore similarly highlighted the vulnerability of heavy social media usage, with nearly 20% of adults at risk of body image anxiety, particularly young females aged 16 to 24 who were most likely to be influenced by celebrities, especially Korean personalities and social media influencers (75, 76). Importantly, research suggests that reducing social media usage can improve body image outcomes. For example, a study by Thai et al. (77) demonstrated that youths who reduced their social media use experienced significant improvements in their perceptions of overall appearance and body weight. Similarly, Smith et al. (78) found that taking a break from social media led to improved body image and self-esteem among young women. These findings highlight that limiting social media use may result in reduced engagement in appearance comparisons and foster healthier self-perception.

However, it is worth noting that social media can also positively impact body image. According to Chang et al. (73), posting selfies was associated with higher body esteem among adolescent girls, indicating that interactions with peers on social media can sometimes promote a positive body image, helping young people gain peer recognition and feel more self-assured. Furthermore, findings from other studies (79, 80) support the idea of social media as a tool to promote body satisfaction among youths. Specifically, the researchers found that exposure to body-positive imagery increased body satisfaction and decreased the drive for thinness in youths.

While BMI and social media use emerged as significant factors across genders, the relationship between childhood maltreatment and body dissatisfaction showed a more complex pattern. Past studies have found that childhood maltreatment is associated with lower body satisfaction in adolescents and young adults (81, 82) (82). For example, research in Germany found that adolescents and young adults with a history of childhood maltreatment showed significantly less satisfaction with their physical appearance than those without such adverse experiences (81). In line with these findings, our study found a significant association between childhood maltreatment and moderate to marked body shape concerns. However, this association was observed only among females, suggesting that there may be gender-specific pathways through which childhood maltreatment affects body image in this population.

According to the identity disruption model proposed by Vartanian et al. (83), early experiences of childhood adversities, which occur in a sensitive developmental period, can interfere with normal identity development, leaving individuals with a less well-defined sense of self. This disruption can make them more susceptible to internalization of body ideals and participation in body-related social comparisons, ultimately contributing to heightened body dissatisfaction. While this model applies to both males and females, the amplified sociocultural pressures on females – including more pervasive exposure to appearance norms and more homogenous and stringent beauty standards – regarding body image (84, 85) likely intensify the impact of childhood maltreatment on their body satisfaction, making them more likely to experience moderate to marked body shape concerns. Conversely, while males also experience body dissatisfaction, the societal expectations and pressures related to body shape and size are generally less pervasive for men, which may buffer the full psychological impact of childhood maltreatment on their body image. Nevertheless, these insights suggest that interventions addressing early trauma may have a particularly strong impact on female youths.

Notably, existing research on gender differences in the psychological mechanisms linking childhood maltreatment and body dissatisfaction remains underexplored. While previous studies have examined associations between childhood maltreatment and body image, research specifically investigating how psychological factors such as self-criticism or self-compassion might differentially mediate this relationship across genders is notably limited. Existing studies have often focused exclusively on young women (86) or among the general population (87) or those with eating disorders without examining potential gender-specific mechanisms (88). Furthermore, the different types and severity of maltreatment were not explored in this study, which could potentially mask important nuances in the relationship between childhood trauma and body dissatisfaction. Future research should investigate how specific forms of childhood maltreatment (e.g., emotional, physical, or sexual abuse) differentially impact body image across genders, and how the severity and duration of traumatic experiences may modulate these effects.

The findings from this study have significant implications for interventions in Singapore. Within educational settings, these interventions could be incorporated into existing school health programs, such as the holistic health framework in primary and secondary schools, which aims to improve students’ overall well-being and promote an active and healthy lifestyle (89). Interactive media literacy education could help youth critically evaluate social media content and beauty standards (90, 91), with these programs extending into post-secondary education institutions to ensure continued support during crucial developmental periods.

From a clinical perspective, targeted interventions are crucial for those experiencing elevated body dissatisfaction, given its comorbidity with mental health conditions such as depression, anxiety, and eating disorders. Attention should be given to individuals with overweight or obesity, focusing on both physical and mental health needs while avoiding weight stigma (92–94). Additionally, trauma-informed approaches may be particularly beneficial for those with childhood maltreatment histories, especially female youths who show increased vulnerability to body image concerns.

At a broader societal level, the findings emphasize the need for culturally relevant and sensitive approaches to addressing body dissatisfaction in Singapore, that consider gender-specific experiences and challenges. Social media literacy programs could help youth balance the potential benefits and risks of social media use, while public health campaigns should promote body diversity and positive body image.

The implementation of these comprehensive interventions could not only improve body image outcomes but also contribute to better overall mental health and well-being among Singapore's youth population. Future research should evaluate the effectiveness of these interventions and continue to identify additional strategies for promoting positive body image in this population.

While this study provides valuable insights into body dissatisfaction among youths in Singapore, it is important to consider both its strengths and limitations. The study benefitted from a large sample size of youths and the use of a validated measure for body dissatisfaction with established cut-off levels. This allowed us to effectively assess the prevalence and severity of body dissatisfaction among young people. However, it is important to consider several limitations when interpreting the findings of this study.

The multiple logistic regression model was adjusted for key sociodemographic variables, including age group, ethnicity, income, and education level, as well as other psychosocial factors such as self-esteem. Therefore, the associations we observed between body dissatisfaction and other predictors like overweight/obesity and social media use already account for these factors. However, it is important to consider that unmeasured factors or potential interactions not included in our model, such as those between age groups and social media use or between overweight/obesity and ethnicity, income, education levels or self-esteem, may still influence the outcomes. Additionally, while the model controls for these variables, the complex nature of body dissatisfaction suggests that other nuanced relationships, such as specific subgroup interactions, may warrant further investigation in future research.

Additionally, the data were cross-sectional, and therefore, temporal associations could not be determined. Discussing body dissatisfaction can be sensitive and potentially distressing for participants, potentially leading to underreporting of true feelings and experiences. However, to mitigate this issue, measures were taken to encourage truthful reporting through assurances of confidentiality and emphasizing that participants’ responses would remain anonymous and would not be linked to their identities. Participants were recruited by convenience sampling; it is possible that individuals with more severe mental health issues may have chosen not to participate in the study due to reluctance to engage in research. This may have led to an underrepresentation of this subgroup, potentially impacting the generalizability of our findings, particularly concerning body dissatisfaction. As a result, the experiences of individuals with severe mental health conditions may not be fully captured in the study.

Despite these limitations, this study adds to the growing understanding of body dissatisfaction by highlighting its associations with sociodemographic and psychosocial factors. Future research should explore the experiences of specific subgroups in more detail.

## Data Availability

Readers who wish to gain access to the data can write to the corresponding author with their requests. Access can be granted subject to the institutional review board (IRB) and the research collaborative agreement guidelines.
